# TNFSF15 and MIA Variant Associated with Immunotherapy and Prognostic Evaluation in Esophageal Cancer

**DOI:** 10.1155/2023/1248024

**Published:** 2023-03-10

**Authors:** Jun You, Jiaojiao Bian, Jian Chen, Tianqin Xia, Ailu Deng, Ming Zhang, YiChen Liao, Huling Wen, Zhengmin Xu

**Affiliations:** ^1^Institute of Medicine, School of Pharmacy, Rheumatic Hematology Department of Affiliated Hospital, Translational Medicine Research Center, Institute of Hepatobiliary Research, North Sichuan Medical College, Nanchong 637000, Sichuan, China; ^2^People's Hospital of Leshan, Leshan 614000, Sichuan, China; ^3^Department of Thoracic Surgery, Rheumatic Hematology Department, Nuclear Medicine Infectious Diseases, Affiliated Hospital of North Sichuan Medical College, Nanchong 637000, Sichuan, China; ^4^Nanchong Hospital of Traditional Chinese Medicine, Nanchong 637000, Sichuan, China; ^5^Cancer Hospital, Affiliated to Medical College of Shantou University, Shantou 515041, China

## Abstract

**Background:**

Esophageal cancer (ESCA) is a common gastrointestinal tumor, and China is one of the regions with a high incidence. Tumor immune-related cells play important roles in the tumorigenesis and development of ESCA. However, the role of tumor immune-related genes in the development of ESCA has not been established.

**Methods:**

In this study, weighted gene coexpression network analysis (WGCNA) was used to analyze ESCA gene expression using data from The Cancer Genome Atlas (TCGA) database. Gene expression was associated with clinical traits, and modules related to CD8+T cells, dendritic cells, and regulatory T cells (Tregs) were obtained.

**Results:**

The GO analysis showed that inflammatory chemotaxis networks were activated by cell chemotaxis, chemokine activity, and chemokine binding receptor. Three hub genes (IL17C, TNFSF15, and MIA) related to tumor immunity and metastasis were identified by WGCNA, and the abnormal expression of each hub gene in ESCA has a poor prognosis, especially in patients with high expression (*P* < 0.05). The risk assessment analysis also showed that tumor stage was positively correlated with tumor risk in ESCA (*P* < 0.05). Therefore, more than 50 pairs of tumor tissues from the T1–T3 stages with different degrees of differentiation and paracancerous tissues were selected to confirm the expression of the three genes using RT-qPCR and immunofluorescence (IF). The infiltration of CD8+ T cells in tumor tissues was lower than that in normal tissues. According to the RT-qPCR, the expressions of IL17 C, TNFSF15, and MIA in moderately and poorly differentiated tissues were significantly higher than those in normal tissues (*P* < 0.05). In contrast, their expressions were decreased in high differentiated tissues (*P* < 0.05). Furthermore, IL17C, TNFSF15, and MIA were all positively correlated with immune checkpoint PD-1; TNFSF15 and MIA were also positively correlated with CTLA4, TIGIT, and CD96.

**Conclusion:**

In summary, IL17C, TNFSF15, and MIA may act as biomarkers for prognosis in moderately and poorly differentiated ESCAs, and they may be used as predictive genes of immunotherapy associated with CD8+ T cell and Tregs invasion in ESCAs.

## 1. Background

Esophageal cancer (ESCA) is one of the most aggressive tumors and the sixth leading cause of cancer-related deaths globally. In developing countries, ESCA ranks eighth in incidence and fifth in mortality [1]. It is expected that the global morbidity and mortality of ESCA will increase within the next few decades [2]. There are two main pathological types of ESCA which are esophageal adenocarcinoma (EAC) and esophageal squamous cell carcinoma (ESCC). Simultaneously radiotherapy and chemotherapy are the main treatment options for advanced ESCA [3]. Due to the limitations of conventional therapies for ESCA, targeted immunotherapy is a key step in the individualized therapy of ESCA [4]. Immunotherapy targeted at programmed death-ligand 1 (PD-L1) or programmed death (PD-1) checkpoints has been approved for ESCA [5, 6]. However, events within the tumor immune microenvironment, including immune cell dysfunction or the accumulation of tumor metabolites, may affect the response of patients with ESCA to immune therapy [7]. As such, the prognosis is still very poor, especially for patients with advanced and metastatic ESCA. Therefore, exploring the mechanisms underlying ESCA immunity and identifying biomarkers is essential for early diagnosis, precision treatment, and prognostic evaluation of cancer.

Tumor-infiltrating immunocytes have been implicated during different stages of tumorigenesis and development [8]. Activated CD8+ T cells play a pivotal role in tumor cytotoxicity, which releases some cytotoxic factors, leading to cell death [9]. Tregs and CD4^+^ FoxP3-T cells (TCONV) are capable of inhibiting antitumor immune responses, promoting tumorigenesis, and accelerating metastasis [10–12]. Tumor-infiltrating immunocytes may affect tumor development and prognosis. In this study, we used WGCNA and GSEA to determine the prognostic markers associated with CD8+ T, CD4+ cells, and Tregs in ESCA. We identified these molecules in 55 ESCA tissues and 55 paired normal adjacent tissues. Furthermore, the cBioPortal database, which includes the clinical and mutation data originating from 2201 patients, was used to analyze hub genes related to overall survival and mutation rates in ESCA. These results will be beneficial for our understanding of the role of CD4^+^ T cells in ESCA and provide a basis for ESCA treatment.

## 2. Methods

### 2.1. Gene Expression Data and Processing

We obtained RNA expression and clinical data of ESCA from TCGA, including those for 165 tumor specimens and 11 normal specimens. First of all, we used the “org.Hs.eg.db” package in R language to convert the “Ensembl id” into a gene symbol (https://www.bioconductor.org). At the same time, we converted RNA-seq-FPKM-count to RNA-seq-TPM-count. Finally, only 1078 immune-related genes downloaded from the ImmPort database (https://www.immport.org/) were selected for WGCNA.

### 2.2. Evaluation of Tumor-Infiltrating Immune Cells (TIICs)

CIBERSORT, a general calculation method, was used to quantify the cellular part from the batch tissue gene expression profiling (GEP). It can accurately estimate the immune components of the tumor biopsies [13]. Therefore, it was used to calculate the proportion of 22 kinds of TIICs in the study, and 10 types of TIICs that were different from normal tissues were selected. The proportion of 5 T-cell subtypes in each sample was selected as the trait data for WGCNA.

### 2.3. GSEA Enriches the Immune-Related Pathways

According to the median of gene expression, we divided the patients into two groups as follows: high and low expression groups. The hallmark, c2, c5, and c7 gene sets downloaded from the Molecular Signatures Database (https://software.broadinstitute.org/gsea/msigdb/index.jsp) were enriched and analyzed using the GSEA_4.1.0. The immune-related pathways were also enriched.

### 2.4. Construction of a Gene Coexpression Network for ESCA

The R software package “WGCNA” is used to construct a coexpression network with the expression values of 1078 genes to generate sample clusters and detect outliers [14]. “FlashClust” was used to screen the samples, and the characters of each sample were transformed into one color. The criterion for selecting the soft threshold is that the degree of freedom is greater than 0.85; therefore, we set the soft threshold to four (scale-free *R*2 = 0.85) to construct the weighted adjacency matrix. Furthermore, the weighted adjacency matrix was transformed into a topological overlap matrix (TOM) to estimate the connectivity of genes in the network generation. Based on the differential detection of TOM, we chose a cutting height of 0.25 and a minimum module size of 30 and divided the gene into nine different modules.

### 2.5. Determination of the Correlation between Modules and Clinical Features

Module eigengenes (MEs) are defined as the principal components of each module. To determine the significance of the modules, we calculated the correlation between the MEs and the level of immune cell infiltration in the tumor tissue using the Pearson test. At *P* < 0.05, an individual module was considered to be significantly related to immune cells. We selected the immune cell subtypes of interest and the module with the highest correlation coefficient and formed the hub module with them. In addition, we calculated the correlation between the genes and clinical features (cor. gene significance) and the correlation between the genes and MEs (cor. Gene module membership) to screen key genes in the hub module.

### 2.6. Multifactor Correlation Analysis and Pearson Correlation Coefficient

We performed multivariate Cox proportional hazards regression analysis to determine the association of clinical parameters, including age, gender, tumor stage, and smoking, with a prognostic assessment of ESCA. Risk scores were calculated using the following formula:

(1) $$\begin{array}{c} \text{risk score}=\sum_{i=1}^{n}{\text{Coefi}}^{*}xi. \end{array}$$

In addition, the correlation between three genes and immune cells was, respectively, analyzed by the Pearson correlation coefficient. Furthermore, the same correlation coefficient was used to analyze the correlation between three hub genes and immune checkpoints. Pearson correlation coefficient was calculated using the following formula:

(2) $$\begin{array}{c} r=\frac{N\sum x_{i}y_{i}-\sum x_{i}\sum y_{i}}{\sqrt{N\sum^{}x_{i}^{2}-{\left(\sum x_{i}\right)}^{2}}\sqrt{N\sum^{}y_{i}^{2}-{\left(\sum y_{i}\right)}^{2}}}. \end{array}$$

### 2.7. Survival and Mutation Analyses

Genes related to inflammation and metabolism were selected as hub genes for the key genes. Next, we used Kaplan–Meier plots (https://kmplot.com/analysis/) to analyze the correlation between the panoncogene (hub gene) mutation group and the normal control group in patients with ESCA. In addition, we obtained the mutation rate of hub genes from the cBioportal database (https://www.cbioportal.org/).

### 2.8. Patient Samples

Fifty pairs of ESCC and adjacent normal tissues were obtained from the affiliated Hospital of North Sichuan Medical College, including high differentiation, middle differentiation, and low differentiation samples. All patients were surgical patients diagnosed with ESCA based on pathology. The tumor stages were based on the eighth edition of the Union for International Cancer Control and American Joint Committee on Esophageal Carcinoma TNM classification system. The clinical characteristics of these patients were obtained from their medical records. The characteristics were age, sex, pathological tumor stage, and degree of tumor differentiation degree (Table 1).

### 2.9. Pathological Hematoxylin-Eosin (HE) Staining and Immunofluorescent Staining

We fixed the collected ESCA tissue as well as paracancerous tissue in 4% paraformaldehyde (PFA) followed by paraffin embedding. The wax blocks were cut into 5 *μ*m sections, partly stained with hematoxylin (Solarbio) and eosin (Solarbio) and partly with immunofluorescence, incubated with CD8 (Bioss) antibodies, and then examined under a fluorescent microscope.

### 2.10. RNA Extraction and Real-Time Quantitative PCR (RT-qPCR)

The tissues were lysed with Trizol reagent (TaKaRa), and the total RNA was extracted. The concentrations of total RNA were detected using a NanoDrop 2000 C spectrophotometer (Thermo Scientific, USA). The total RNA was reverse-transcribed into cDNA by Revertaid First Strand cDNA synthesis reagent (CAT: ^#^K1622, Thermo Fisher Science). The cDNAs were amplified by real-time quantitative PCR (RT-qPCR) with specific primers (Table 2), and PowerUp SYBR Green Master Mix (CAT: ^#^A25742, Thermo Fisher Science) was used as the luminescent substrate. Finally, we analyzed the melting curve, and the expression of the hub gene was normalized with *β*-actin.

## 3. Results

### 3.1. Infiltration of Mature T and B Cells in ESCA Decreased While Naïve B Cells Increased

**Figure 1:**
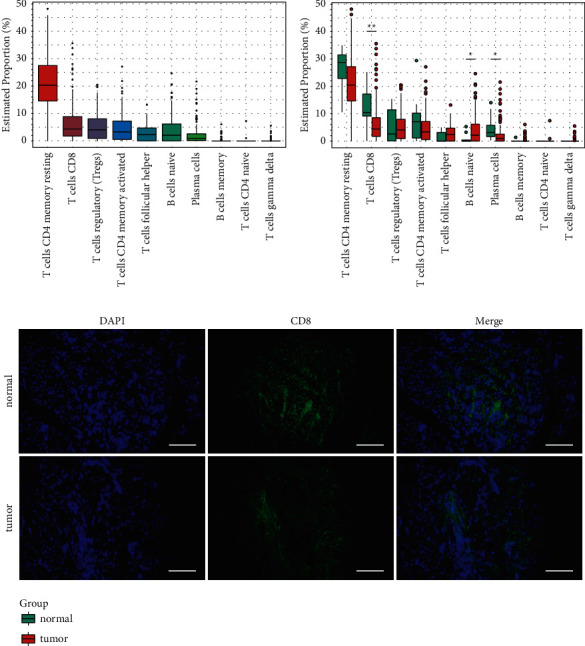
Distribution of immune infiltrating cells in tumors and its difference from that for normal tissue. (a) The percentage of ten kinds of adaptive immune-related cells. (b) The proportion of immunocytes in tumor and normal tissues. (c) Immunofluorescence staining of CD8 in tumor tissue and its paracancerous tissues.

The analysis of adaptive immune cell infiltration by 10 subtypes in 161 ESCA samples showed that the proportion of memory resting CD4 T cells in the quiescent stage was the highest, followed by those of the CD8 T cells, memory activated CD4 T cells, follicular helper T cells, naïve B cells, and plasma cells (Figure 1(a)). The differences in immune infiltrating cells between the normal and tumor samples were compared and analyzed, and we discovered that the degree of immune cell infiltration differed between normal and tumor tissues. The degree of infiltration of CD8 T cells and plasma cells in the tumor was significantly lower than that in normal tissues (*P* < 0.05), while the infiltration of naïve B-cells in the tumor was higher than that in the normal group (*P* < 0.05) (Figure 1(b)). As shown in Figure 1(c), the CD8 T cells were significantly reduced in tumors compared to paracancerous tissues.

### 3.2. Analysis of Immune-Related Pathways Using GSEA

**Figure 2:**
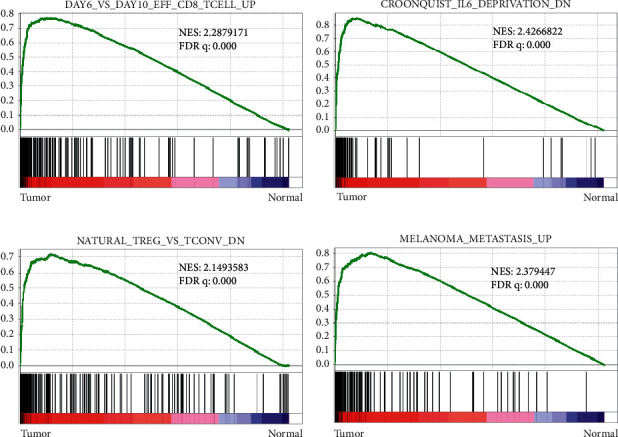
Analysis of immune-related pathways by GSEA. (a–d) Differences between tumor immune infiltration- and tumor metastasis-related pathways of normal and tumor tissues based on the FDR *q* value of <0.25. (a) Effector CD8 T-cell. (b) IL6 deprivation. (c) Natural Treg. (d) Melanoma metastasis.

GSEA was used to analyze the differences in tumor immune infiltration and tumor metastasis-related pathways for the normal and tumor tissues. As shown in Figure 2(a), the degree of enrichment of characteristic genes of effector CD8+ T cells during the early stage of cancer was remarkably higher than that during the later stage (*P* < 0.05); the downregulated IL6 deprivation pathway genes were enriched in tumor tissues (*P* < 0.05) (Figure 2(b)), CD4^+^FoxP3-T (TCONV) cell characteristic genes that promote tumor migration and metastasis were enriched in tumors, while nature Treg cell characteristic genes were downregulated (Figure 2(c)), and melanoma metastasis characteristic genes promoting the tumor metastasis pathway were enriched in tumor tissues (Figure 2(d)).

### 3.3. Weighted Coexpression Network of ESCA

**Figure 3:**
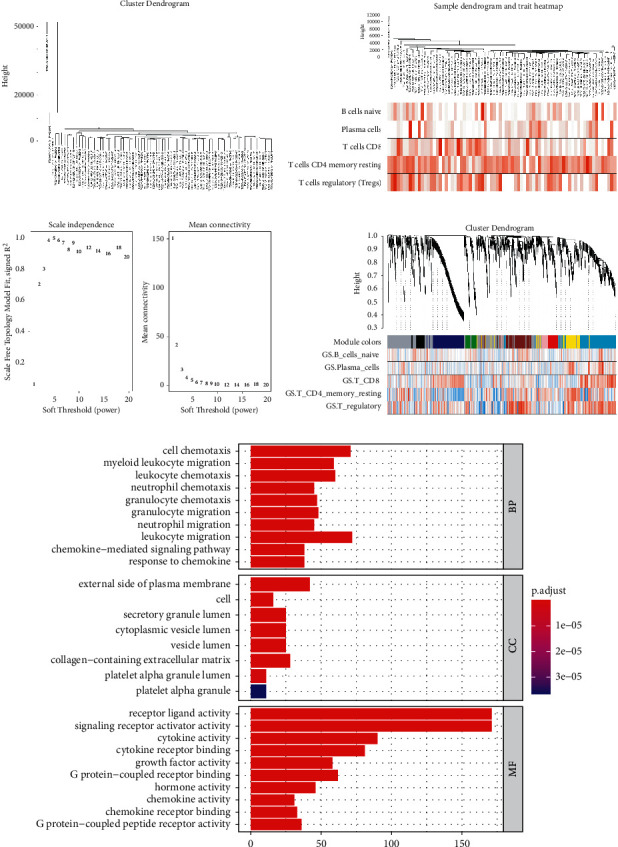
Outlier elimination and analysis of network topology using a diverse soft-thresholding power to construct a scale-free network. (a) Sample cluster tree: the threshold was 30000, and there was one outlier sample. (b) The sample dendrogram and trait heatmap after deleting the outlier samples. (c) The scale-free fit index of the 1–20 soft threshold power (*β*) is analyzed. (d) Genes are divided into different modules by hierarchical clustering, and different colors represent different modules.

We obtained 1100 gene expression matrices and clinical information related to adaptive immunity in ESCA tissues from the TCGA database, excluding one outlier sample, and constructed a hierarchical clustering tree (Figure 3(a)) for the remaining 76 samples. In addition, we labeled the immune cells with the greatest infiltration in cancer tissues according to the immune landscape in the ESCA. Besides, we observed significant differences from normal tissues under the resulting tree (Figure 3(b)). Following the proposal of the file package, the soft threshold power value was used to construct a gene coexpression network (Figure 3(c)). Based on the construction of the coexpression module clustering and hierarchical clustering trees, a total of nine modules were identified (Figure 3(d)).

### 3.4. Correlation between Modules and Adaptive Immune Cells with Significant Differences in Tumor Tissues

**Figure 4:**
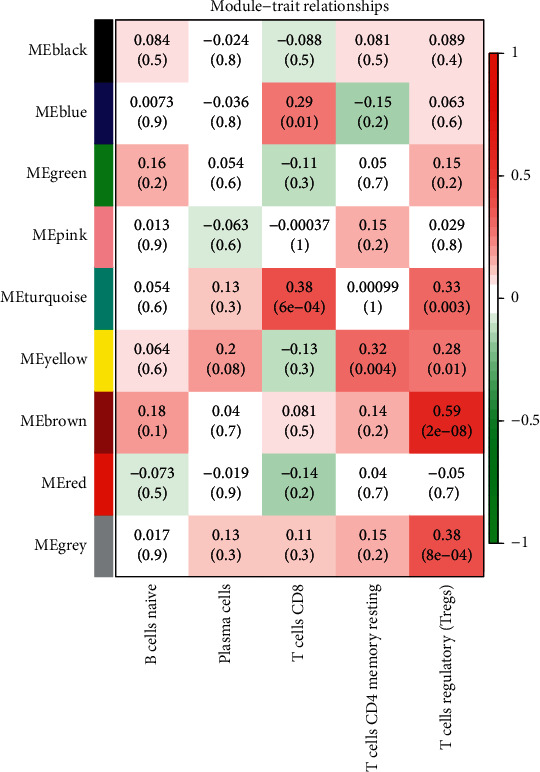
Module-trait relationships. The Pearson correlation coefficients for module eigengenes and immunocytes infiltration.

We determined the coexpression genes of each module according to their gene characteristics and calculated the correlation of the characteristic value of each module based on the characteristics of the adaptive immune cells, the cells with higher correlation are CD8^+^ T cells, naïve B cells, plasma cells, memory resting CD4 T cells, and regulatory T cells. We selected two modules with the highest correlation in the CD8^+^ T cells, as denoted by the ME-turquoise (*R*2 = 0.38, *P* < 0.05) and ME-blue rows (*R*2 = 0.29, *P* < 0.05). At the same time, we selected two modules with the highest correlation in the regulatory T cells, as denoted by the ME-turquoise (*R*2 = 0.33, *P* < 0.05) and ME-brown (*R*2 = 0.59, *P* < 0.05) rows (Figure 4). The bubble chart shows similar results (Supplementary Figure 1).

### 3.5. Three Hub Genes Were Genetic Variants of ESCA

**Figure 5:**
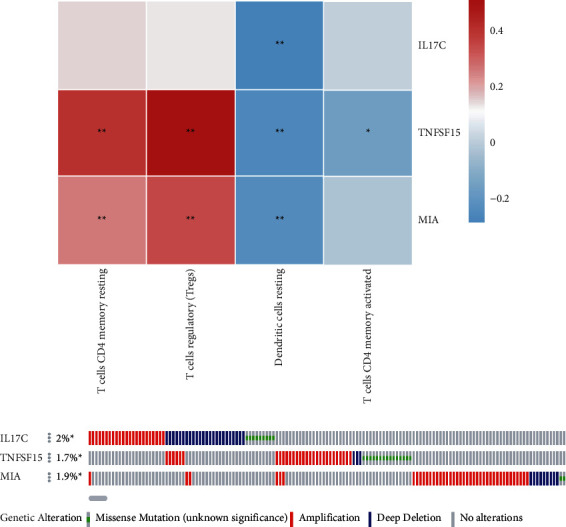
The hub genes are the key to genetic variation in ESCA. (a) The relationship between hub genes and immune-related cells. (b) Genetic variation analysis of three hub genes using the cBioportal database.

Based on WGCNA analysis, the expression of three hub genes IL17C, TNFSF15, and MIA were all negatively associated with dendritic cells. Among them, TNFSF15 expression was also negatively associated with CD4^+^ memory-activated T cells. In addition, TNFSF15 and MIA expression were positively associated with Tregs and CD4^+^ memory resting T cells, respectively (*P* < 0.05) (Figure 5(a)). In addition, the genetic variability of these genes in ESCA was analyzed. The analysis of genetic variation in the cBioPortal database showed that the proportion of IL17C, TNFSF15, and MIA in ESCA was 2.0%, 1.7%, and 1.9%, respectively (*P* < 0.05), and they all presented several alterations including amplification and deletion in patients with ESCA (Figure 5(b)).

### 3.6. TNFSF15, IL17C, and MIA Are Important Biomarkers for Evaluating the Prognosis of Patients with ESCA

**Figure 6:**
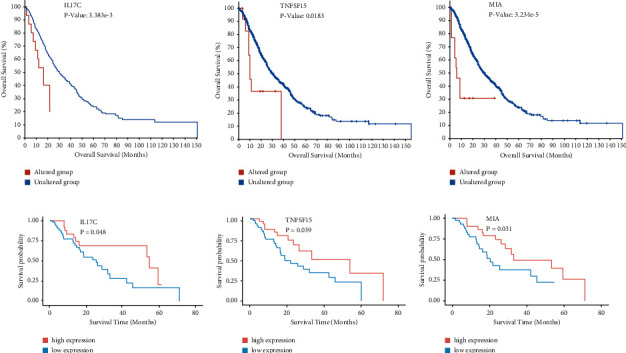
Survival analysis of IL17C, TNFSF15, and MIA. (a–c) Overall survival analysis of biomarker genes from cBioportal; red represents the altered group and green represents the unaltered group, *P* < 0.05; (d–f) survival time of patients with high or low expression of IL17C, TNFSF15, and MIA; *P* < 0.05.

We further evaluated the value of the above hub gene selection in evaluating the prognosis of patients with ESCA, and the overall survival (OS) of the patients with the three abnormal hub genes was analyzed using the cBioPortal database. The cBioPortal database contains 2201 clinical samples from eight ESCA-related research websites. As shown in Figure 6(a)–6(c), the patients with ESCA with abnormalities of IL17C, TNFSF15, and MIA had remarkably lower 5-year and 10-year survival rates than the controls, and their overall survival duration was not more than five years (*P* < 0.05). The overall survival duration of patients with IL17C gene abnormalities was shorter, even less than one year (*P* < 0.05). Moreover, we also analyzed the relationship between the expression of the three genes and prognostic survival using the TCGA database. The results showed that patients with low expression of IL17C, TNFSF15, or MIA lived longer than those patients with high expression of one of them (*P* < 0.05) (Figure 6(d)–6(f)).

### 3.7. IL17C, TNFSF15, and MIA Expression Correlated with the Degree of Differentiation of ESCA, Validated in Tissues from Stage T1 to T3 Esophageal Squamous Cell Carcinoma

**Figure 7:**
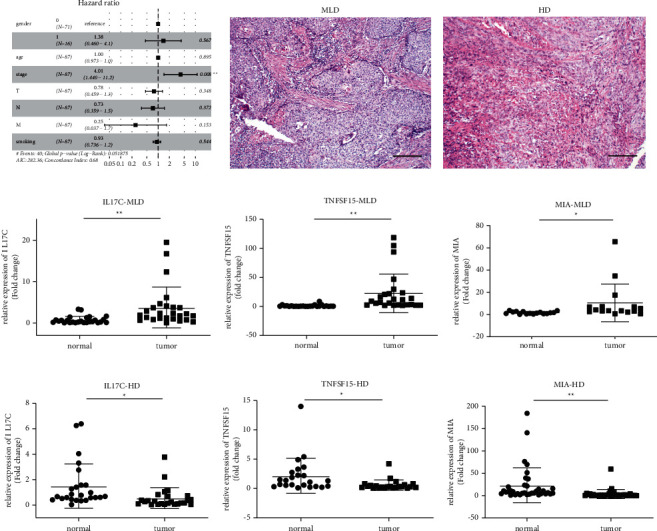
Histopathological staining of ESCA at different stages and relative mRNA expression of 3 hub genes in ESCA tissues and paracancerous tissues based on Graphpad. (a) Middle and low differentiated. (b) High differentiated. (c–e) The expression of IL17C, TNFSF15, and MIA in MLD. (f–h) The expression of IL17C, TNFSF15, and MIA in MLD. Normal: normal tissues; tumor: esophageal squamous cell carcinoma; MLD: middle and low differentiated, HD: high differentiated. ^*∗*^*P* < 0.05; ^∗∗^*P* < 0.01. The scale bar is 100 *μ*m.

Some clinical parameters: age, gender, stage, and smoking are prognosis evaluations in the clinical. As shown in Figure 7(a), there was a positive correlation between the ESCA stage and prognosis evaluation (*P* < 0.05). Therefore, tissues from patients with stage-T1 to T4 ESCA who were only treated with surgery were selected to verify these candidate genes. The tumor stage was determined based on the results of pathological HE staining (Figures 7(b) and 7(c)). Then, the qPCR assay was performed for 55 pairs of clinical samples, including adjacent and tumor tissues. As shown in Figure 7, the expressions of IL17C, TNFSF15, and MIA in tumor tissues were significantly different from those in adjacent tissues. In tissues with moderate and low differentiation, the expressions of these three genes were significantly higher than those in tumor-adjacent tissues (*P* < 0.05) (Figures 7(d)–7(f)). In highly differentiated ESCA tissues, by contrast, the expressions of IL17C, TNFSF15, and MIA in tumor tissues were significantly lower than those in normal tissues (*P* < 0.05) (Figures 7(g)–7(i)).

### 3.8. Relationship between the Three Genes and Immune Checkpoints in ESCA

**Figure 8:**
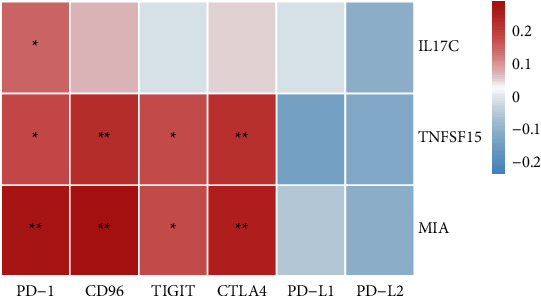
Hub genes in ESCA are related to immune checkpoints. ^*∗*^*P* < 0.05; ^∗∗^*P* < 0.01.

PD-1, CTLA4, CD96, and TIGIT are important immune checkpoints that are potential targets for tumor immunotherapy. To further understand the role of these genes in ESCA, the relationship of the three genes with those immune checkpoints was assessed. As suggested in Figure 8, MIA and TNFSF15 expression were significantly positively correlated with PD-1, CTLA4, CD96, and TIGIT in ESCA (*P* < 0.05); IL17C expression was positively correlated with PD-1 (*P* < 0.05) (Figure 8).

## 4. Discussion

CD8+ T cells play an essential role in effective immune responses. In our study, the infiltration of cytotoxic T cells was decreased in tumor tissues, suggesting that tumor immunity was disregulated. Furthermore, CD4^+^FoxP3-T (TCONV) cell characteristic genes that promote tumor migration and metastasis were enriched in tumors, while nature Treg cell characteristic genes were downregulated. Therefore, to determine the characteristics of adaptive immunity-related genes in ESCA, we performed RNA-seq analysis of the ESCA samples and compared the expressions of adaptive immunity-related genes with that of normal tissues, and three modules related to CD8 T cells and Tregs were obtained.

On this basis, IL17C, TNFSF15, and MIA which were related to tumor immunity, invasion, and metastasis were identified. IL17C, an autocrine cytokine, is an important factor in the innate immunity of the epithelium. IL17C may promote or inhibit tumorigenesis by regulating immune cell function [15–17]. In our clinical samples, it was amplified in moderately and poorly differentiated tissues but downregulated in high differentiated tissues. The survival duration of patients with altered IL17C was less than 30 months, and patients with high expression of that were living shorter than those with low expression. TNFSF15 belongs to the tumor necrosis factor (TNF) ligand family induced by TNF and IL-1*α*, which plays an important role under disease conditions, such as cancer and stroke, by maintaining vascular and lymphatic vessel homeostasis [18–21]. In our study, the expression of TNFSF15 was significantly higher in moderately and poorly differentiated tissues than in normal tissues, and patients with TNFSF15 alterations were in poor prognosis. All this suggested that TNFSF15 may play a risk role in moderate and low differential ESCA. MIA is a neurotransmitter involved in tumor invasion and migration. MIA is a melanoma-derived growth regulatory protein that can inhibit the growth of melanoma cells and some other neuroectodermal tumors (including gliomas) in vitro, and it may be an antitumor molecule in melanoma [22]. But in ESCAs, the expression of MIA was increased in moderately or poorly differentiated tumor tissues, and patients with high differentiated tumors lived longer than that middle-low differentiated patients, which indicates that MIA may have different roles according to the different tumor. All this suggests that IL17C, TNFSF15, and MIA may be prognostic risk factors for OS in middle-low differentiated ESCA, respectively.

Furthermore, the analysis of immunocyte infiltration in ESCA suggested that CD8+ T cells were significantly lower than that in paracancerous tissue, and we used IF to confirm that CD8+ T cells were reduced in ESCA tissues. PD-1, CTLA4, CD96, and TIGIT are associated with each other in tumor immunity, which are candidates for immunotherapy [23–26]. IL17C and TNFSF15 are involved in tumorigenesis and development by impacting immune cell function, and MIA is associated with tumor invasion [27–29]. Then, we analyzed the relations among IL17C, TNFSF15, and MIA and the immune checkpoint. TNFSF15 and MIA were positively associated with the four immune check-points, PD-1, CTLA4, CD96, and TIGIT. In addition, IL17C was only positively associated with PD-1 expression, and we used IF to confirm that IL17C was highly expressed in ESCA tissue. These results indicate that highly expressed IL17C, TNFSF15, and MIA in ESCA may influence the development and prognosis of ESCA through participating in the immune regulation process, which may act as predictors of immunotherapy for ESCA.

However, there were some limitations in our study, such as the sample size limiting the accuracy of the analysis and the experimental results. Furthermore, more time was needed to review patients with special gene phenotypes and evaluate their prognosis. Finally, the specific mechanisms underlying the actions of these alteration genes in ESCA need to be elucidated in future research.

In summary, we used multiple biological information analyses and experiments involving clinical samples to identify potential biomarkers of ESCA. Three hub genes were identified to be abnormally expressed in tumors, and they affected the prognosis of patients with ESCA. The immune cell function regulating IL17C, TNFSF15, and MIA have been identified as potential biomarkers for the evaluation of ESCA diagnosis and prognosis, which may also be predictors of immunotherapy.

## 5. Conclusions

In this study, CD8+ T cells were decreased in tumor tissues which contributed to the immune imbalance of the tumor. WGCNA was used to identify the characteristics of immune-related genes in ESCA. Three modules related to CD8+ T cells and Tregs were identified, and three hub genes related to the survival duration and prognosis of ESCA were obtained, which are IL17C, TNFSF15, and MIA, and they can be used as therapeutic targets for further study and clinical markers to predict the prognosis of patients.

## Figures and Tables

**Table 1:** Patient characteristics.

Variables	Total patients, *N* = 55	Degree of differentiation		
		High, 20	Middle, 24	Low, 70
Age, *y*, mean ± SD	67.93 ± 8.5	65.8 ± 8.7	70.17 ± 6.5	67.43 ± 9.7

Sex				
Male	42 (76.36)	17 (85)	18 (75)	5 (71.43)
Female	13 (23.64)	3 (15)	6 (25)	2 (28.57)

Pathological stage				
I	8 (14.55)	2 (10)	4 (16.67)	0
II	24 (43.64)	12 (60)	7 (29.17)	4 (57.14)
III	13 (54.17)	3 (15)	8 (33.33)	2 (28.57)
IV	3 (5.45)	1 (5)	2 (8.34)	0

**Table 2:** RT-qPCR primer sequence.

The primer	F (5′-3′)	R (3′-5′)
IL17C	CCACACTGCTACTCGGCTG	CACACGGTATCTCCAGGGTGA
TNFSF15	CACCACATACCTGCTTGTCAGC	TCTCCGTCTGCTCTAAGAGGTG
MIA	GCCAAGTGGTGTATGTCTTCTCC	CTGGTCCTCTCGGACAATGCTA

## Data Availability

Publicly available datasets were used in this study. The data can be found in the TCGA database and the cBioportal website.

## Abbreviations

ESCA: Esophageal cancer
WGCNA: Weighted gene coexpression network analysis
TCGA: The Cancer Genome Atlas
IF: Immunofluorescence
EAC: Esophageal adenocarcinoma
PD-L1: Programmed death-ligand 1
PD-1: Programmed death
TCONV: Tregs and CD4+ FoxP3-T cells
GEP: Gene expression profiling
TIICs: Tumor-infiltrating immune cells
TOM: Topological overlap matrix
MEs: Module eigengenes
PFA: Paraformaldehyde
HE: Hematoxylin-eosin
RT-qPCR: Real-time quantitative PCR
OS: Overall survival.

## Supplementary Materials

Supplementary Materials Supplementary Figure 1: scatter plots of gene significance and module membership. Supplementary Figure 2: the boxplot of 9 biomarker genes for gene expression data in TCGA.
