# Supplementary material for: TNFSF15 and MIA Variant Associated with Immunotherapy and Prognostic Evaluation in Esophageal Cancer
Source: J Oncol. 2023 Mar 10;2023:1248024. doi: 10.1155/2023/1248024 (PMC10023233; doi:10.1155/2023/1248024)
Supplement: Supplementary Materials — Supplementary Figure 1: scatter plots of gene significance and module membership. Supplementary Figure 2: the boxplot of 9 biomarker genes for gene expression data in TCGA. [file 1248024.f1.zip › Supplementary Figure 1.pdf]

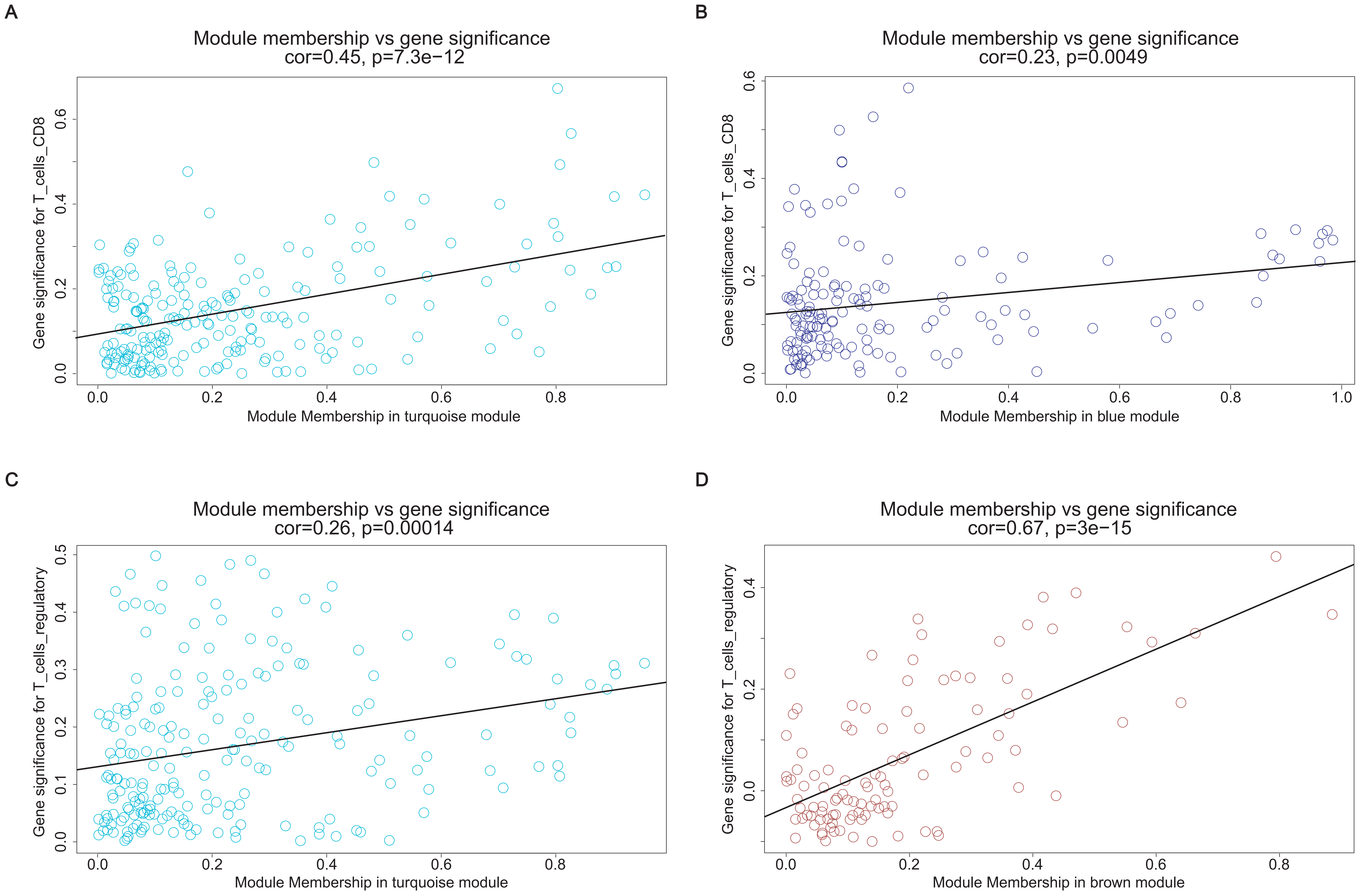
A
Module membership vs gene significance
cor=0.45, p=7.3e−12
Gene significance for T_cells_CD8
Module Membership in turquoise module
B
Module membership vs gene significance
cor=0.23, p=0.0049
Gene significance for T_cells_CD8
Module Membership in blue module
C
Module membership vs gene significance
cor=0.26, p=0.00014
Gene significance for T_cells_regulatory
Module Membership in turquoise module
D
Module membership vs gene significance
cor=0.67, p=3e−15
Gene significance for T_cells_regulatory
Module Membership in brown module
