# Supplementary figures and images for: TNFSF15 and MIA Variant Associated with Immunotherapy and Prognostic Evaluation in Esophageal Cancer
Source: J Oncol. 2023 Mar 10;2023:1248024. doi: 10.1155/2023/1248024 (PMC10023233; doi:10.1155/2023/1248024)

A

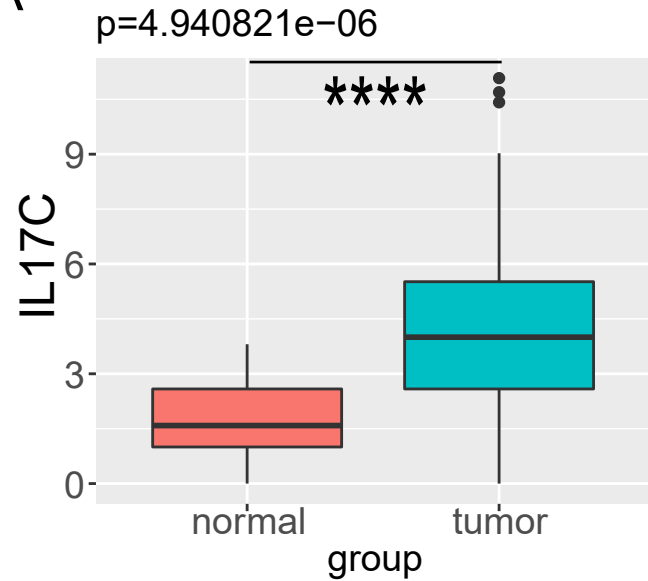

B

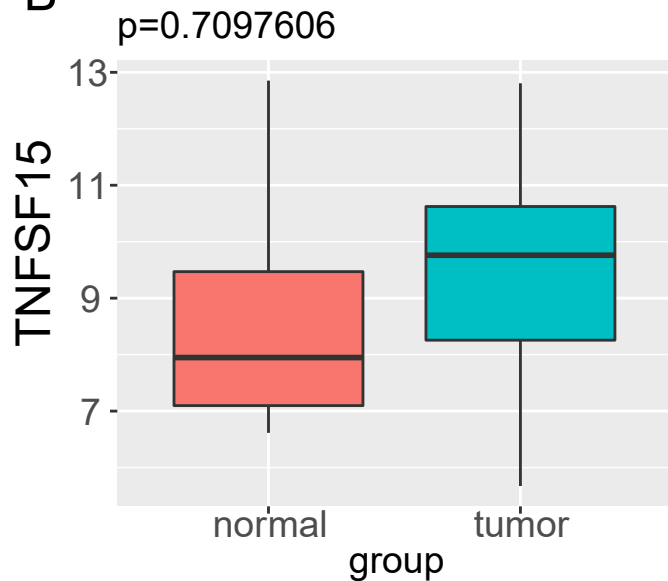

C

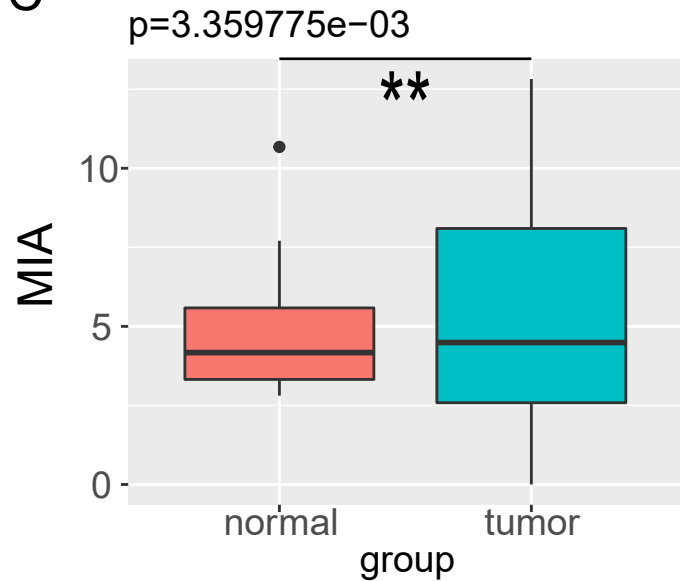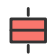

normal

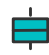

tumor

Supplement: Supplementary Materials — Supplementary Figure 1: scatter plots of gene significance and module membership. Supplementary Figure 2: the boxplot of 9 biomarker genes for gene expression data in TCGA. [file 1248024.f1.zip › Supplementary Figure 2.pdf]
